# Prognostic utility of cardiovascular magnetic resonance upright maximal treadmill exercise testing

**DOI:** 10.1186/s12968-015-0208-z

**Published:** 2015-11-25

**Authors:** Bunyapon Sukpraphrute, Brandon C. Drafts, Pairoj Rerkpattanapipat, Timothy M. Morgan, Paul M. Kirkman, William O. Ntim, Craig A. Hamilton, Robert L. Cockrum, W. Gregory Hundley

**Affiliations:** Departments of Internal Medicine (Cardiovascular Medicine Section), Wake Forest Health Sciences, Medical Center Boulevard, Winston-Salem, North Carolina NC 27157-1045 USA; Public Health Sciences, Wake Forest Health Sciences, Winston-Salem, North Carolina USA; Biomedical Engineering, Wake Forest Health Sciences, Winston-Salem, North Carolina USA; Department of Radiology, Wake Forest Health Sciences, Winston-Salem, North Carolina USA; Frye Heart Center, Hickory, North Carolina USA; Mid Carolina Cardiology, Charlotte, North Carolina USA

**Keywords:** Coronary artery disease, Exercise testing, Cardiovascular magnetic resonance, Stress, Prognosis

## Abstract

**Background:**

Left ventricular wall motion abnormalities (LVWMA) observed during cardiovascular magnetic resonance (CMR) pharmacologic stress testing can be used to determine cardiac prognosis, but currently, information regarding the prognostic utility of upright maximal treadmill induced LVWMA is unknown. Our objective was to determine the prognostic utility of upright maximal treadmill exercise stress CMR.

**Methods:**

One hundred and fifteen (115) men and women with known or suspected coronary arteriosclerosis and an appropriate indication for cardiovascular (CV) imaging to supplement ST segment stress testing underwent an upright treadmill exercise CMR stress test in which LVWMA were identified before and immediately after exercise. Personnel blinded to results determined the post-test incidence of cardiac events (cardiac death, myocardial infarctions [MI], and unstable angina warranting hospital admission or coronary arterial revascularization).

**Results:**

All participants completed the testing protocol, with 90 % completing image acquisition within 60 s of exercise cessation. MI or cardiac death occurred in 3 % of individuals without and 17 % of individuals with inducible LVWMA (*p* = 0.024). The combination of MI, cardiac death, and unstable angina warranting hospitalization occurred in 14 % of individuals without and 47 % of individuals with inducible LVWMA (*p* = 0.002). The addition of CMR imaging identified those at risk for future events (*p* = 0.002), as opposed to the electrocardiogram stress test alone (*p* = 0.63).

**Conclusions:**

In patients with or suspected of coronary arteriosclerosis and appropriate indication for imaging to supplement ST segment analysis during upright treadmill exercise, the presence of inducible LVWMA during treadmill exercise stress CMR supplements ST segment monitoring and helps identify those at risk of the future combined endpoints of myocardial infarction, cardiac death, and unstable angina warranting hospitalization.

## Background

Left ventricular wall motion abnormalities (LVWMA) identified during pharmacologic stress cardiovascular magnetic resonance (CMR) forecast future cardiac events including myocardial infarction (MI) and cardiac death [1–6]. Although interpretation of CMR after pharmacologic stress is utilized in patients unable to walk on a treadmill, exercise as a stress agent is preferred over pharmacologic stress in those individuals who can walk or ride a stationary bicycle [7–9]. At present, due to the need for rapid image acquisition and the logistics of locating a treadmill close to the CMR scanner, relatively few CMR stress studies have been performed after treadmill exercise. As a consequence, the feasibility of utilizing CMR after treadmill exercise to detect inducible LVWMA and forecast cardiac prognosis is largely unknown.

The objective of this study was to determine the prognostic utility of CMR assessments of stress induced LVWMA obtained immediately after upright maximal treadmill exercise. To address this objective, we performed stress imaging using rapid CMR acquisition techniques immediately after upright maximal treadmill exercise in patients with chest pain that exhibited an appropriate indication for treadmill exercise with adjunctive imaging [7–9]. Personnel blinded to the results of stress testing ascertained the post-stress test occurrence of cardiovascular events.

## Methods

### Study design

The Institutional Review Board at the Wake Forest University School of Medicine approved this study. The design included a retrospective analysis of participant outcomes after receiving an upright treadmill exercise test. All participants provided written, informed consent (for CMR) and concurrent medical record review. During the longitudinal study, personnel without access or familiarity with the CMR stress test results obtained additional consent obtained. This included verbal consent with the participant or family member to identify any hospitalizations and an additional written consent to review their respective medical and hospital records for the cause of any hospitalization, or permission to obtain copies of death certification. The study population consisted of 115 men and women with known or suspected coronary artery disease (CAD). Between April 2002 and July 2009, each participant received an upright maximal treadmill exercise CMR stress test to identify inducible LVWMA indicative of myocardial iscemia. Patients with contraindications to CMR (implanted pacemakers, defibrillators, intracranial metal, or claustrophobia) or upright treadmill exercise were excluded from enrollment [10]. Thirty-five of the 115 subjects were also participants of a prior study by Rerkpattanapipat et al., who evaluated the diagnostic utility of this form of testing [11].

### Stress test protocol

**Fig. 1:**
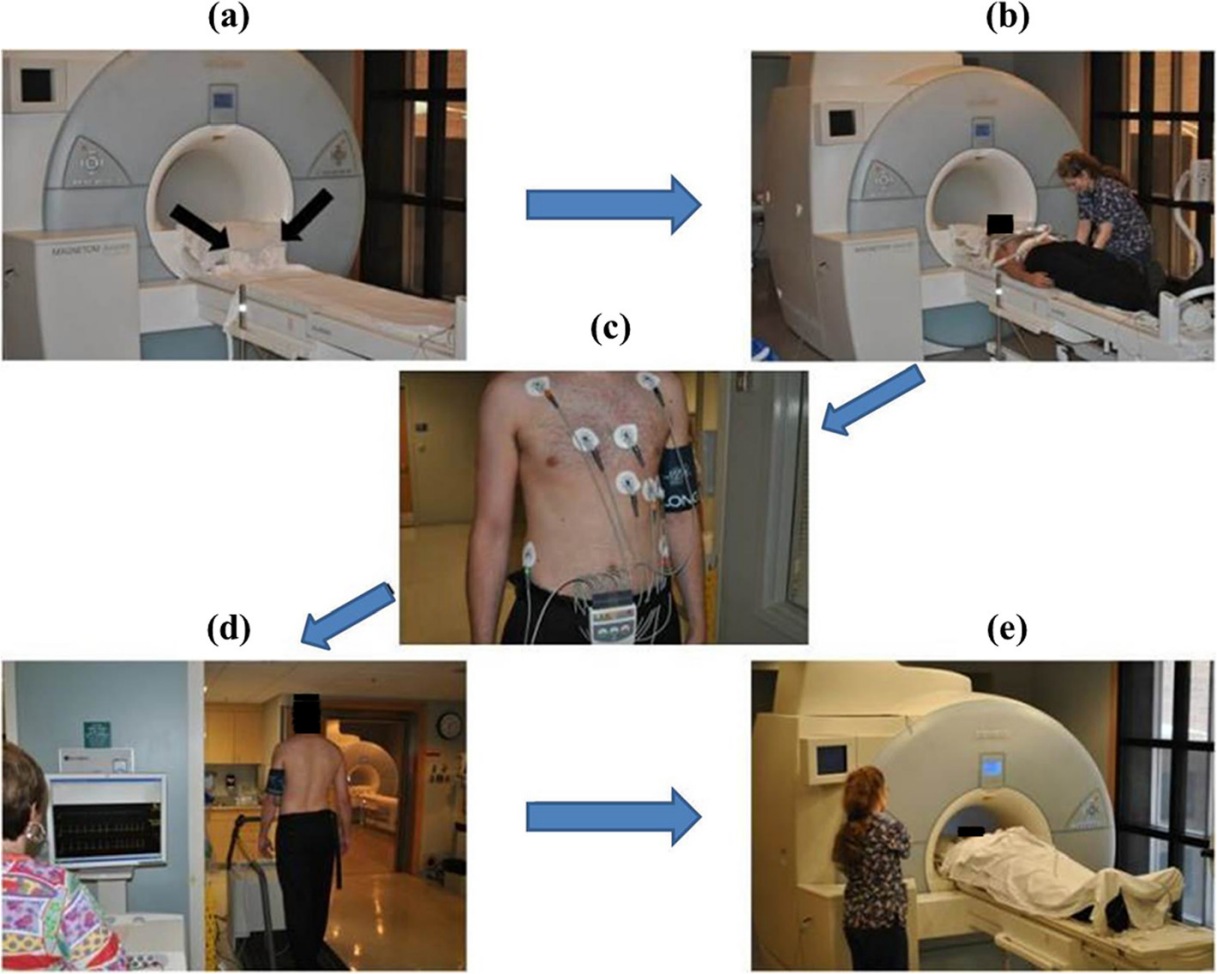
Upright Treadmill Exercise CMR Stress Test Protocol. CMR scanner with shoulder harnesses (*black arrows*) to aid with correct patient positioning (**a**), obtaining rest cine imaging (**b**), placement of electrode patches and blood pressure cuff for patient monitoring (**c**), treadmill exercise stressing (**d**), and repositioning the patient into the scanner for post-stress cine images (**e**)

The stress test protocol is displayed in Fig. 1. Shoulder harnesses on the table enabled correct positioning of the participant and ensured that the participant returned to the same position after exercise. Non-metallic electrodes were placed on the participant and a supine resting electrocardiogram (ECG) was performed. After this ECG was obtained, resting cine imaging was acquired with each participant lying supine on the CMR table within the bore of the scanner.

Participants were then escorted to a treadmill that was placed outside of the CMR room approximately 20 ft from the end of the CMR table. All ferromagnetic components of the treadmill except the motor were refitted with stainless steel or aluminum equivalents. An upright resting 12-lead ECG was obtained. Each participant exercised using a Bruce or modified Bruce protocol with a pre-test goal during exercise to achieve 80 % of the maximum predicted heart rate response for age, a response previously established for dobutamine stress CMR for identifying ischemia and forecasting cardiac prognosis [12, 13].

While exercising, the staff maintained constant communication with the participant to determine the point at which the participant felt they were in the final 30 s of exercise. At this point, the staff completed a peak exercise upright 12-lead ECG and transferred the participant to the CMR scanner, using the shoulder harnesses for correct repositioning for stress imaging. Exercise was also terminated if the participant had complaints of angina or ST-segment depression ≥ 1.5 mV. A final recovery supine ECG was obtained following the protocol.

Imaging was acquired with the following two types of CMR scans at 1.5 T with a cardiac surface coil laid over the chest. All participants were imaged using a prospective ECG-gated, breath-held acquisition by acquiring 3 slices of 8 mm thickness in short-axis view (base, mid, apex; so that all 16 myocardial segments were visualized) with a 192 × 144 matrix, a 340 × 255 mm field of view, a 3.8 ms temporal resolution, a 1.3msecho time, a 50 flip angle, a 40 cm field of view, and 14 to 18 views per segment, (resulting in a temporal resolution of 46 ms and a voxel size of 1.8 × 1.8 × 8 mm [11]).

With the advent of “real-time” or “snap-shot” imaging, the last 80 participants also (after the breath-hold scan) underwent a 5 to 10 s real-time, non-gated, free breathing acquisition with the following parameters: 6–10 slices of 8 mm thickness and 2 mm gap spanning the entire left ventricle in short-axis view, a 128x72 matrix, a 340 × 255 mm field of view, 72 views per segment with view sharing, and echo spacing of 2.1 ms, resulting in a temporal resolution of 86 ms and voxel size of 2.6 × 2.6 × 8 mm.

**Fig. 2:**
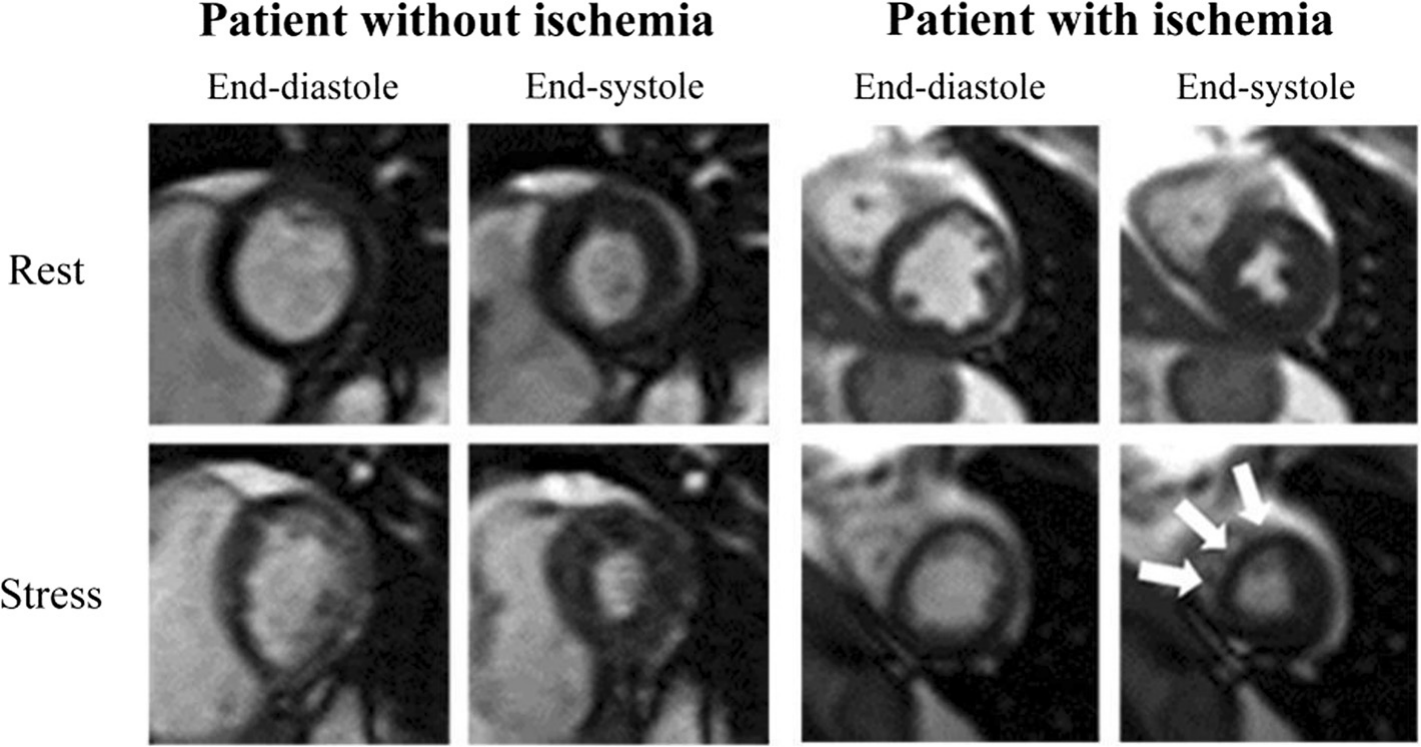
Rest and Stress CMR Images of the Left Ventricle in the Short-Axis View. Cine CMR images of the left ventricle in the mid-short axis view at end-diastole and end-systole during rest (*top row*) and after maximal upright treadmill exercise stress (*bottom row*) in participants without (*left*) and with (*right*) treadmill exercise-induced left ventricular wall motion abnormality (LVWMA). White arrows mark an area of inadequate thickening of the left ventricular myocardium indicative of an inducible LVWMA associated with myocardial ischemia

Inducible ischemia was evaluated on both the breath-hold and real-time acquisition independently by personnel blinded to the result of the other image acquisition through assessing LVWM in 16 myocardial segments at rest and after stress [10, 14–16]. During rest, post exercise, and recovery, LV regional wall motion was labeled as normal, hypokinetic, akinetic, or dyskinetic, and LVWMA were defined as deterioration in regional wall motion within a myocardial segment after exercise relative to wall motion observed during rest imaging (Fig. 2). In all participants, left ventricular ejection fraction (LVEF) was calculated at rest using a multi-slice short axis modified Simpson’s rule technique [17].

### Outcome data

All patients were contacted by personnel blinded to the stress testing results to determine the post-exercise incidence of cardiac events. Hard events were defined as cardiac death (death due to acute coronary syndrome, cardiac arrhythmia, or congestive heart failure) or non-fatal myocardial infarction (rise in cardiac biomarkers with at least one value exceeding the 99^th^ percentile with either angina symptoms or new ST-T segment changes) [18]. Other events included unstable angina (anginal chest pain with non zero admission serum biomarker of myocardial injury without pronounced ST segment elevation [19]) warranting hospital admission, and in some of the same cases, coronary artery revascularization [20–22]. If more than one cardiac event was discovered during follow-up, the worst event was used for analysis- according to the following paradigm: cardiac death > MI > unstable angina. All reported events were obtained by a questionnaire and then verified after a thorough review of the patient’s medical records. In the case of a participant’s death, an immediate family member’s consultation and death certificate were used for confirmation.

### Statistical analysis

Patients were categorized according to the presence or absence of inducible LVWMA indicative of ischemia from their treadmill exercise CMR study. All grouped data were expressed as mean ± standard deviation. Group differences for categorical variables were tested with the Fisher’s exact test. A multivariable step-wise logistic regression model was used to identify independent predictors of events, with *p* < 0.05 as the inclusion and exclusion levels. The relative risk of having an event for a given variable was expressed by a hazard ratio (HR). Variables were considered significant if the null hypothesis of no contribution could be rejected at a probability value of < 0.05. The probability of the presence or absence of cardiac events as a function of follow-up duration was estimated by the Kaplan-Meier method and compared between groups by use of the log-rank test. A power analysis was conducted for the log-rank test comparing time to event rates between the ischemic and non-ischemic events. The analysis showed that this cohort would provide 80 % power to detect a hazard ratio of 3.2 for any cardiac event.

## Results

All 115 participants completed the upright treadmill exercise CMR stress testing protocol. At rest and after stress, 1825 (99 %) and 1828 (also 99 %) of the 1840 myocardial segments were visualized with the breath-hold technique. One participant developed supraventricular tachycardia during exercise that resolved within two minutes of rest. Ventricular fibrillation, MI, or death did not occur during stress testing. One hundred of the 115 participants achieved 80 % of the maximum predicted heart rate response (MPHRR) for age during exercise.

Demographic data and a summary of cardiac events are displayed in Table 1. When compared to the group without inducible LVWMA, the participants with inducible LVWMA exhibited more hypertension and hyperlipidemia (*p* = 0.003 to 0.004). Hemodynamic data from the participants are shown in Table 2. Peak stress caused an average increase in heart rate of 58 bpm and systolic blood pressure of 20 mmHg at an average metabolic equivalents (METS) of 9.

**Table 1:** Demographic data and summary of events

	ALL	Reached 80 % target HR or ischemia	Inducible LVWMA	No Inducible LVWMA	*p*-value
Patient characteristics					
Patients	*n* = 115	*n* = 100	*n* = 30	*n* = 70	
Age, yrs	59 ± 13	59 ± 13	62 ± 12	58 ± 14	0.17
Gender men	71 (62)	59 (59)	21 (70)	38 (54)	0.18
BMI, kg/m2	28.4 ± 5.9	28.1 ± 5.9	29.4 ± 6.4	27.5 ± 5.5	0.13
LVEF, %	58 ± 10	58 ± 10	56 ± 11	59 ± 9	0.091
Historical Information					
Prior MI	29 (25)	24 (24)	11 (37)	13 (19)	0.073
Hypertension	63 (55)	51 (51)	22 (73)	29 (41)	0.004
Hyperlipidemia	74 (64)	61 (61)	25 (83)	36 (51)	0.003
Diabetes	24 (21)	20 (20)	9 (30)	11 (16)	0.11
Smoking	35 (30)	35 (35)	8 (27)	19 (27)	1.00
Events					
All Cardiac Events	30 (26)	24 (24)	14 (47)	10 (14)	0.002
Cardiac Death / MI	8 (7)	7 (7)	5 (17)	2 (3)	0.024

Values are expressed as n (%), unless otherwise indicated

*BMI* body mass index, *LVEF* left ventricular ejection fraction, *LVWMA* left ventricular wall motion abnormalities, *MI* myocardial infarction

**Table 2:** Hemodynamic data

	All	Reached 80 % target HR or ischemia	Inducible LVWMA	No Inducible LVWMA	*p*-value
Resting HR (beats/min)	78 ± 14	78 ± 13	76 ± 14	80 ± 13	0.36
Peak HR (beats/min)	136 ± 21	161 ± 13	158 ± 12	162 ± 14	0.17
% MPHRR	88 ± 12	91 ± 11	87 ± 14	93 ± 6	0.009
METS	9 ± 3	9.5 ± 3.4	8.0 ± 3.1	10 ± 3	0.004
Resting SBP (mm Hg)	138 ± 23	139 ± 23	144 ± 20	137 ± 24	0.23
Peak SBP (mm Hg)	158 ± 23	158 ± 23	164 ± 25	155 ± 22	0.20
Resting DBP (mm Hg)	74 ± 12	75 ± 12	75 ± 8	75 ± 13	0.89
Peak DBP (mm Hg)	81 ± 9	81 ± 9	76 ± 9	83 ± 9	0.18

*DBP* diastolic blood pressure, *HR* heart rate, *MPHRR* maximum predicted heart rate response, *SBP* systolic blood pressure; other abbreviations as in Table 1

Since there are no current appropriateness statements for upright treadmill exercise CMR stress testing, we examined the appropriateness of each individual to receive imaging (ultrasound or radio-isotope testing) to supplement ST segment monitoring during exercise according to American College of Cardiology Statements for these other modalities [7–9, 23]. Each participant’s history, past cardiac studies, clinical presentation, and resting ECG were reviewed independently. Of the 115 participants, 55 % had baseline ECG abnormalities, 18 % had a history of poor acoustic windows with echocardiography, 8 % had a prior equivocal work-up, and 5 % had a recent positive treadmill exercise ECG stress test. As such, all 115 participants were deemed appropriate for imaging testing using Appropriateness criteria for these other noninvasive cardiac imaging modalities [7–9, 23].

After the treadmill exercise CMR study, contact was made with all participants after an average of 3.7 ± 2.6 (median 2.6, range 0.6 to 7.8) years. There were 7 total hard events which included one cardiac death (Table 3). Five participants suffered a non-cardiac death without any prior cardiac events. The results of the ECG portion of each stress test were also collected to determine the utility of the ECG stress result for assessing prognosis. Thirty-five participants developed inducible ischemia indicated by ≥ 1 mm of ST segment depression [8], with 9 of these 35 experiencing inducible LVWMA by CMR after their exercise. The overall effect of exercise-induced significant ST segment depressions on prognosis was not significant for any cardiac event or MI and cardiac death (*p* = 0.63 and 0.08 respectively). The 3-year event rates in participants with abnormal treadmill exercise ECG studies was 37 % versus 24 % with a normal ECG result (*p* = 0.89).

**Table 3:** Cardiac events (expressed as total number of those achieving 80 % MPHRR for age and percent)

	*n*
Unstable angina warranting hospitalization	11 (11)
Coronary artery revascularization	
PCI	6 (6)
CABG	0 (0)
Myocardial infarction	6 (6)
Cardiac death	1 (1)

*PCI* percutaneous coronary interventions

*CABG* coronary artery bypass graft

*MPHRR* maximum predicted heart rate response

Thirty of the 100 subjects experienced inducible LVWMA after the upright treadmill exercise CMR stress test. In those with and without inducible LVWMA, the overall proportion of participants with a) any cardiac event was 47 and 14 %, respectively (*p* = 0.002; Table 1), and b) MI or cardiac death was 17 and 3 %, respectively (*p* = 0.024).

When all variables in Table 1 were considered in the multivariable step-wise logistic regression model, CMR evidence of inducible LVWMA and the presence of hypertension were independently associated with any cardiac event (*p* = 0.012 and < 0.001, respectively). Inducible LVWMA, prior MI and a history of smoking were independently associated with MI or cardiac death (*p* = 0.01 to 0.04). After controlling for inducible LVWMA, the patient’s age, BMI, LVEF, or history of prior MI, diabetes, or hyperlipidemia were not associated with an increase in the odds ratio of any cardiac event (*p* = 0.06 to 0.99 for all).

**Fig. 3:**
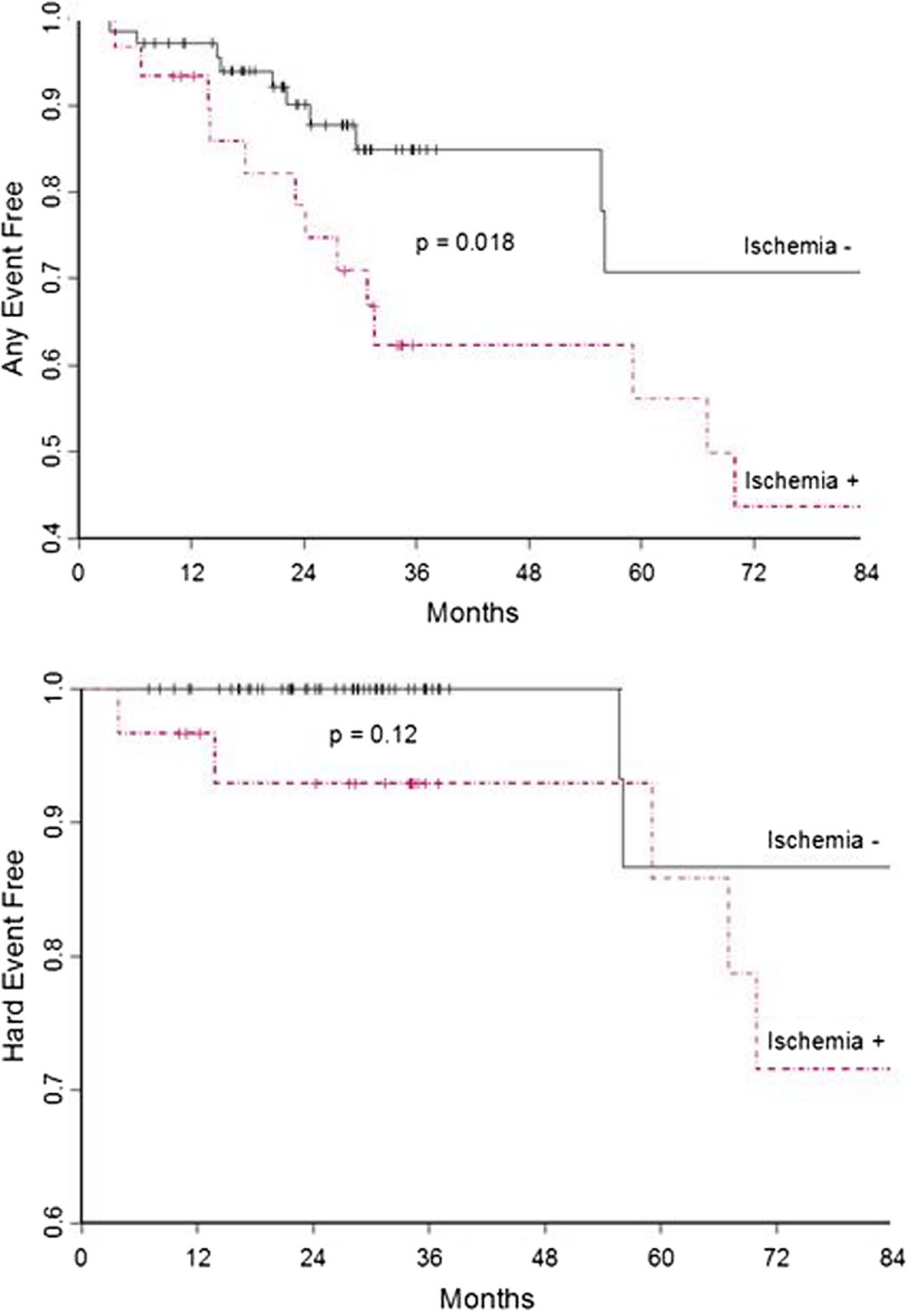
Kaplan-Meier Survival Plots. Survival plots showing the proportion of participants free from cardiac events (any cardiac events, *top*; hard cardiac events, *bottom*) versus time. Participants without inducible ischemia are indicated by red dashed lines and those with inducible ischemia are indicated by the black solid lines

A Kaplan-Meier analysis showing the proportion of participants free from any and hard cardiac events is shown in Fig. 3. The hazard ratio for those with compared to those without inducible LVWMA is 2.08 for any cardiac event (*p* = 0.08) and 3.30 for MI or cardiac death (*p* = 0.02). The 3-year event free rate for MI or cardiac death for the participants without inducible LVWMA was 100 %, compared to 92.9 % of the participants with inducible LVWMA (*p* = 0.072).

Overall, with the breath-holding method, 38 out of 115 participants (33 %) exhibited a resting LVWMA abnormality, and 47 out of 115 participants (41 %) exhibited a stress LVWMA. In this study, 80 individuals underwent both real time and breath hold cine wall motion imaging at rest and after peak stress. At rest, 41 % of the studies exhibited normal wall motion in both real time and breath hold imaging. Twenty-five percent of images exhibited abnormal wall motion with real time and breath hold images. The other 34 % of individuals either displayed normal wall motion with the real time technique and abnormal wall motion with the breath hold technique, or vice versa. With stress, there was more concordance between the real time and breath hold techniques. Both techniques displayed normal wall motion in 62 % of individuals, and abnormal wall motion in 16 % of individuals. Abnormal wall motion with one or the other technique with normal wall motion in the other technique was present in only 12 % of cases. There was greater agreement in blinded wall motion assessments with stress (*p* = 0.006).

The log rank test comparing stress induced LVWMA indicative of ischemia for time to cardiac event gave a *p*-value of 0.0179 when ischemia was determined using breath hold techniques, and a *p*-value of 0.0176 when ischemia was identified using the real time techniques. The method used to define ischemia appeared to give equal prognostic significance for identifying those at risk of experiencing future cardiovascular events.

## Discussion

The results of this study indicate the following: (1) it is feasible to perform CMR wall motion assessments in a timely fashion immediately after upright maximal treadmill exercise in patients with or suspected of coronary artery disease; (2) the results of treadmill exercise CMR stress LV wall motion assessments can be used to forecast major adverse cardiac events; (3) the addition of CMR imaging provides additional information as to who will or will not experience adverse cardiac events after upright maximal treadmill exercise when the resting 12-lead ECG is abnormal.

The present study is the largest to date that examines the feasibility of utilizing upright maximal treadmill exercise stress testing in combination with CMR cine assessments of LV wall motion to assess cardiac prognosis. Although pharmacologic stress has been used with CMR imaging to identify LV myocardial ischemia, viability, and cardiac prognosis in patients who are incapable of adequate physical exercise, relatively few studies have been performed with exercise stress. When possible, exercise stress testing is preferred, as one can assess exercise capacity and associate physical activity with patient symptoms [15]. In this study, all 115 participants completed the protocol and LVWM was assessed in 99 % of the LV myocardial segments at rest and within 60 s after completion of exercise.

The stress ECG portion of the test did not forecast future cardiac events in our study. This is likely due to the majority of our study population experiencing ECG abnormalities at baseline (54 %), which are known to decrease the diagnostic accuracy of exercise induced ECG changes [24]. Compared to ECG analyses alone when the resting 12-lead ECG is abnormal, the addition of echocardiography or nuclear scintigraphy to treadmill exercise stress testing provides higher sensitivity and specificity for diagnosing inducible ischemia [25]. Our results indicate that the addition of CMR imaging to ECG monitoring allows one to identify those both at and not at risk for future cardiac events.

Appropriateness criteria for exercise stress testing coupled with CMR imaging have yet to be described. Each participant in our study was originally referred for evaluation of myocardial ischemia and underwent an upright treadmill exercise CMR stress test for assessment. All cases were reviewed and found to be appropriate according to the latest appropriateness guidelines for stress echocardiography, and cardiac radionuclide imaging [7–9, 23]. These criteria took into account the pre-test probability of CAD, the ability to exercise, uninterpretable baseline ECG, and prior equivocal stress tests. The results of this study may contribute to further consideration for developing CMR exercise stress criteria. When considering the development of these criteria, it is important to recognize that this study was performed in a state-of-the-art facility with CMR technologists, nursing staff, exercise physiologists and cardiovascular medicine specialists trained in CMR safety, stress study performance and image interpretation.

Four prior studies have reported on protocols for acquiring CMR images after upright treadmill exercise stress testing. Rerkpattanapipat, et al., assessed 35 patients with chest pain using treadmill exercise CMR stress testing and found a sensitivity and specificity of 79 and 85 %, respectively, of inducible LVWMA for identifying > 70 % coronary artery luminal diameter narrowing on contrast coronary angiography [11]. This preliminary finding was encouraging given that the majority of the patients exhibited > 70 % luminal narrowing in a single coronary artery (often termed “single vessel coronary artery disease”), a condition that may be more difficult to identify than multi-vessel disease [26]. Subsequently, Jekic, et al., successfully used CMR in 20 volunteers without CAD to measure cardiac function and myocardial perfusion at peak stress after maximal exercise on a non-ferromagnetic treadmill inside the MRI room located just beyond the 2 Gauss line [27]. While 100 % of our participants completed diagnostic imaging within 60 s using a treadmill outside of the CMR room, an additional 10 s could be gained by placing the treadmill in the same room as the MRI scanner as in the study used by Jekic, et al. A study by Thavendiranathan, et al. compared exercise CMR to echocardiography in 28 healthy volunteers, examining left ventricular endocardial wall visualization. They found that a greater proportion of the segments were visualized by CMR [28]; in our study 99 % of our participants’ LV myocardial segments were visualized. Finally, a study by Raman, et al. explored real time cine and myocardial perfusion with treadmill exercise stress CMR in individuals referred for stress SPECT imaging (single photon emission computed tomography). A follow up exam at 6 months indicated freedom from cardiovascular events in 29/29 CMR negative individual, while participants in our study without inducible LVWMA had a 2 % chance in 3 years of experiencing an event [29].

The presence of an inducible LVWMA was associated with a higher rate of future cardiac events, including MI and cardiac death (Table 1). Results from the multivariable step-wise logistic regression analysis showed that inducible LVWMA were independent predictors of future cardiac events, including MI or cardiac death, after adjusting for hypertension for any cardiac event and smoking history for hard events.

Our findings are similar to the results of studies that evaluated the prognostic value of other noninvasive stress imaging techniques in patients with known or suspected CAD. In this study, the three year event-free survival rate for MI or cardiac death for participants without inducible LVWMA on treadmill exercise CMR stress testing is 99–100 %, which is consistent with < 1 % event-free survival rate of MI or cardiac death per year for the first three years in studies on dobutamine stress CMR, adenosine stress CMR, and nuclear perfusion tests [1–6, 30]. Participants with inducible LVWMA on treadmill exercise CMR stress testing had a 1-year event rate of 5 % (Fig. 3), which is similar to patients with inducible ischemia on exercise stress echocardiography, dobutamine stress radionuclide scintigraphy, or dobutamine stress CMR (1-year event rates of 7 to 11 %) [1, 31, 32].

Rapid, “real-time” cine imaging became available in the latter years of this study. As such, 80 individuals underwent both breath-hold and real-time assessments of LV wall motion before and after exercise stress. Using either form of LV wall motion analyses, one was able to forecast cardiac prognosis. Relative to the baseline or pre-exercise assessments of LV wall motion, the concordance between distinguishing abnormal versus normal wall motion using the blinded analyses of the two (breath-hold versus real time) forms of imaging was improved after exercise stress (*p* < 0.0001).

## Conclusions

Our study has the following limitations. First, the study population included primarily Caucasian participants, as only 8 % were African-American. Thus results apply primarily to Caucasians and further studies may be required in those of other races or ethnicities. Second, myocardial perfusion imaging was not performed. Studies have demonstrated the additional value of myocardial perfusion imaging with pharmacologic CMR stress testing [33], and future developments in exercise CMR stress testing protocols could potentially allow for the assessment of LVWM and myocardial perfusion during the same imaging sequence. Third, we performed a retrospective follow-up of the participant outcomes. While our results were able to forecast major adverse cardiac events, there was only a strong trend toward forecasting myocardial infarction and cardiac death. Studies with larger numbers of cardiac events could be performed to identify the precision of this methodology and identify those at risk of infarction or cardiac death. Finally, we did not assess LV wall motion assessments after (or during) bicycle exercise. Bicycle exercise ergonometers are now available for attachment to MR tables and future studies are necessary to evaluate the prognostic utility of this modality.

In conclusion, in adequately equipped centers with trained personnel, upright treadmill exercise CMR stress testing is feasible and these retrospective results suggest this may serve as a noninvasive alternative study for the assessment of cardiac prognosis in adults who are able to walk on the treadmill and do not possess contraindications to CMR.
